# Primary erythromelalgia: a review

**DOI:** 10.1186/s13023-015-0347-1

**Published:** 2015-09-30

**Authors:** Zhaoli Tang, Zhao Chen, Beisha Tang, Hong Jiang

**Affiliations:** Department of Neurology, Xiangya Hospital, Central South University, 87 Xiangya road, Changsha, 410008, Hunan China; Key Laboratory of Hunan Province in Neurodegenerative Disorders, Central South University, 87 Xiangya road, Changsha, 410008, Hunan China; State Key Lab of Medical Genetics, Central South University, 110 Xiangya road, Changsha, 410078, Hunan China

**Keywords:** Primary erythromelalgia, Voltage-gated sodium channel, Pain, Genetics, Electrophysiology, Hyper-excitability, Therapy

## Abstract

Primary erythromelalgia (PE ORPHA90026) is a rare autosomal dominant neuropathy characterized by the combination of recurrent burning pain, warmth and redness of the extremities. The incidence rate of PE ranges from 0.36 to 1.1 per 100,000 persons. Gender ratio differs according to different studies and no evidence showed a gender preference. Clinical onset of PE is often in the first decade of life. Burning pain is the most predominant symptom and is usually caused and precipitated by warmth and physical activities. Reported cases of PE contain both inherited and sporadic forms. Genetic etiology of PE is mutations on *SCN9A*, the encoding gene of a voltage-gated sodium channel subtype Nav1.7. Diagnosis of PE is made upon clinical manifestations and screening for mutations on *SCN9A*. Exclusion of several other treatable diseases/secondary erythromelalgia is also necessary because of the lack of biomarkers specifically for PE. Differential diagnoses can include Fabry disease, cellulites, Raynaud phenomenon, vasculitis and so on. Diagnostic methods often involve complete blood count, imaging studies and thermograph. Treatment for PE is unsatisfactory and highly individualized. Frequently used pain relieving drugs involve sodium channel blockers such as lidocaine, carbamazepine and mexiletine. Novel drugs such as PF-05089771 and TV-45070 could be promising in ameliorating pain symptoms due to their Nav1.7 selectivity. Patients’ symptoms often worsen over time and many patients develop ulcerations and gangrenes caused by excessive exposure to low temperature in order to relieve pain. This review mainly focuses on PE and the causative gene *SCN9A --* its mutations and their effects on Nav1.7 channels’ electrophysiological properties. We propose a genotype-channelopathy-phenotype correlation network underlying PE etiology which could provide guidance for future therapeutics.

## Introduction

Pain, under physical conditions, informs body of harmful stimuli, elicits protective reflexes, making it essential for survival. Pathological pain, on the other hand, is one of the most prevalent symptoms seen in patients. Chronic pain has become a global health problem which is estimated to affect about 30 % of adults worldwide [1]. Currently used drugs such as opioids and non-steroidal anti-inflammatory drugs (NSAIDs) show limited efficacy [2]. Thereby more precise medications for various types of pain rising from different etiologies are required. As one of the human heritable pain disorders, primary erythromelalgia (PE) is characterized by the triad of recurrent burning pain, warmth and redness of the extremities. The causative gene for PE, *SCN9A*, encodes a voltage-gated sodium channel (VGSC) subtype Nav1.7. Series of researches on PE have elucidated a close relationship between aberrant electrophysiology of VGSC (in this case Nav1.7) and hyperexcitability of peripheral nociceptive neurons. PE plays a pivotal role in understanding the molecular mechanisms underlying pain caused by neuron hyperexcitability. This review mainly focuses on PE and the causative gene *SCN9A* -- its mutations and their effects on Nav1.7 electrophysiological properties. We also recapitulate utilization of current treatments and updates of novel Nav1.7-targeted agents, and propose an integrated perspective of a genotype-channelopathy-phenotype network underlying this intricate condition.

### Definition

PE is a rare autosomal dominant neuropathy characterized by the triad of burning pain, recurrent redness, and warmth of the extremities.

### Nomenclature and classification

This rare and debilitating disorder was first reported and named “erythromelalgia” according to its main symptoms (erythros-red, melos-limbs, algos-pain) by Mitchel in 1878. Later the name “erythromelalgia” and “erythermalgia” were used respectively for cases secondary to myeloproliferative disorders and idiopathic cases that were refractory to aspirin. At present, erythromelalgia is classified into PE and secondary erythromelalgia, based on the absence or presence of diseases or drugs that may associate with the onset or precipitation of its symptoms [3, 4]. PE is exclusively caused by mutations in *SCN9A*, the encoding gene of sodium channel subtype Nav1.7 and can be sub-classified into familial (inherited erythromelalgia) and sporadic forms. Secondary erythromelalgia is often associated with myeloproliferateive disorders. In some cases, secondary erythromelalgia occurs in paraneoplastic diseases and autoimmune neuropathies. Though very rare, secondary erythromelalgia can also appear in diabetes, rheumatologic diseases and infectious diseases [5–9].

### Epidemiology

PE is a rare neuropathy. Little is known in terms of the global prevalence of PE. In a Norwegian clinical study of 87 patients with Erythromelalgia, the estimated annual prevalence was 2/100,000 with primary cases making up two-thirds of all cases [10]. A recent study in Sweden reported that the incidence of erythromelalgia was 0.36/100,000 though no incidence data regarding primary form was shown [11]. In a population-based study in Olmsted County, USA, the incidence was reported 1.3/100,000. Male-to-female ratio of erythromelalgia differs greatly in the aforementioned studies [12]. However, these studies included both primary and secondary forms of erythromelalgia, therefore providing limited information about prevalence/incidence of PE. Patients reported present diverse nationalities (Chinese, American, French, Dutch, Norwegian etc.) and geographical backgrounds [13–36].

### Clinical description

PE is characterized by the hallmark triad of recurrent redness, burning pain, and warmth of the extremities in virtue of exposure under heat, exercise and gravity and can be relieved by cooling and elevation [37]. Clinical onset of PE often takes place by the first decade but can also display a broad range from congenital to early 60s [19, 38]. Warmth, ambulation, physical exercise, sitting, leg dependence and wearing shoes or gloves can provoke and aggravate PE symptoms while immersion in ice/cold water, uncovering affected areas, ventilation and elevation can ameliorate these symptoms [39]. In some cases the symptoms precipitated during puberty, implicating a hormonal role in PE pathophysiology [40].

Pain is the most disturbing symptom affecting PE patients and can be disabling. The pain is usually bilateral, intermittent and severe, more often involving lower extremities than upper extremities [39]. Besides extremities, body parts such as hands and auricles being affected have been reported [41]. Pain attacks usually start with an itchy-like feeling and then progress to a severe burning sensation as was reported by most patients, with the durations of which ranging from several minutes to hours and even days. Pain symptoms are worse in summer and at night and are usually provoked and exacerbated by heat, ambulation, physical exercise, sitting, leg dependence, and coverage of extremities [40]. Cooling and elevation are most effective ways to relieve pain symptoms. According to patients, they would immerse affected limbs in ice water, uncover their feet during sleep or walk barefoot in winter. Affected extremities can develop ulceration and gangrene which are not directly attributable to PE but are results of excessive exposure to low temperature [42].

Apart from the common clinical descriptions, there have been several reports about atypical PE cases with comorbidities. Takahashi et al. described a PE patient with wintry hypothermia and encephalopathy [43]. Meijer et al. reported a case of PE patient with global motor decay [14].

### Etiology

#### Genetics

The causative gene for PE, *SCN9A* (GenBank: DQ148960.1), was first identified and reported by Yang et al. in 2004 [36]. *SCN9A* encodes Nav1.7, a member of the VGSC family. Nav1.7 is preferentially expressed in small-diameter nociceptive neurons and can be up-regulated in the context of inflammation [44]. It is now well characterized that Nav1.7 plays a pivotal role of “threshold gate” in the generation of action potentials for its ability to amplify small, slow depolarizations and thus bringing the membrane potential closer to the voltage threshold of action potential [45]. Due to the strong dependence of the action potential on Nav1.7 in nociceptive neuron cells, molecular alterations in Nav1.7 caused by *SCN9A* mutations can lead to nociceptive dysfunction. More than 70 mutations on *SCN9A* have been associated with various clinical phenotypes, among which are pain disorders including gain-of-function disorders PE and paroxysmal extreme pain disorder (PEPD) as well as loss-of-pain disorder congenital insensitivity to pain (CIP) [36, 46, 47]. To date, more than 20 mutations have been reported responsible for PE [13–36].

#### Sodium channel and Nav1.7

**Fig. 1:**
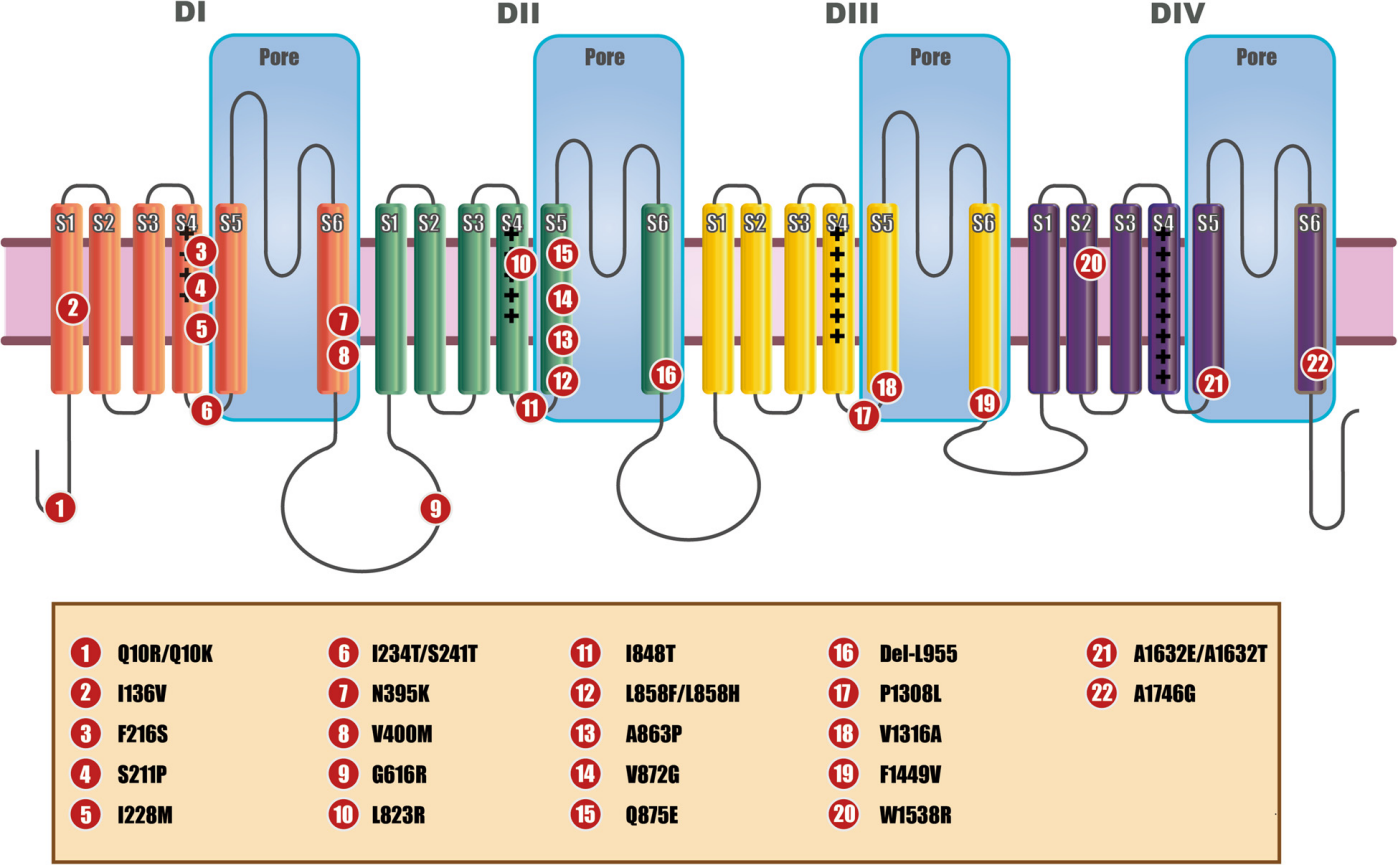
Schematic of voltage-gated sodium channel alpha subunit and localization of reported PE mutations. The alpha subunit consists of four domains (I-IV). Each domain contains six helical transmembrane segments (S1-S6). The S4 segment of each domain contains positively charged amino acid residues

Action potentials are triggered by noxious stimuli and then propagated along the pain axis from peripheral to central nervous system. During this process, VGSCs play a pivotal role [48]. Nine subtypes of VGSCs, Nav1.1-Nav1.9, have been identified in mammals, each holding distinguishable features and interacting with other VGSC members in an intricate manner [49]. Analogous to other VGSC subtypes, Nav1.7 is an integral membrane protein that consists of a large alpha subunit and auxiliary beta subunit(s) [50]. The alpha subunit forms the voltage-sensitive and ion-selective pore, which predominantly determines neural electrophysiological characters (Fig. 1). The ion permeation pore is formed by proximal deploy of the 4 domains within the alpha subunit [51]. More specifically, the S1-S4 segments are considered voltage sensing structures, where positive amino acids in S4 segments act as gating charges that react to electrical field alteration and confer conformation changes of the permeation pore, thereby forming permeation pathways for Na + currents [52]. Previous studies have demonstrated that the S4 segments in domain I and II determine voltage dependence of activation, while those in domain III and IV contribute to coupling activation and inactivation [53]. Particularly, the DIIIS4/S5 linker sequence is highly conserved among sodium channels, suggesting a conserved role in channel function [23]. Close to the outer surface of the channel is the narrowest part of the pore, where the linkers that connect S5 and S6 segments form the locus of ion selectivity [54], thereby mutations within this locus could lead to sodium channelopathies [18]. In addition, S6 segments in domain III and III-IV linkers have been proofed crucial to fast inactivation [35, 55]. These regions also contain drug-binding spots involved in inactivation [51, 54, 56].

Nav1.7, the first sodium channel identified as a contributor to chronic pain [57], produces rapid activation and inactivation currents that are sensitive to sub-micromolar levels of tetrodotoxin (TTX) [58]. Nav1.7 is an important component in the peripheral nociceptors, as has been proofed in series of studies on its gating properties, syndromes associated with its mutations and behavioral tests in knock-out mice [45, 59]. Nav1.7 is also characterized by slow closed-state inactivation through which its own depolarizations (ramp currents) can be activated in response to small slow depolarizations close to resting potentials [60–62]. Thus Nav1.7 channels have the capacity of amplifying subtle depolarizations and play the part of “threshold gate” in nociception pathways [45], by virtue of escalating the membrane potential towards the activation threshold of Nav1.8 channels, which generate all-or-none action potentials [63, 64]. The sophisticated interaction between Nav1.7 and Nav1.8 may elucidate divergent electrophysiology of dorsal root ganglia (DRG) and sympathetic neurons [45].

#### SCN9A mutations and neuron excitability

*SCN9A*, located on chromosome 2q31-32 [65], is a highly polymorphic gene, with more than 70 mutations residing within it associated with various clinical phenotypes [17]. Yang et al. first narrowed down the candidate gene of PE to VGSC genes, and identified 2 mutations (L858H and I848T) residing in *SCN9A.* Since then more than 20 PE mutations have been identified (Table 1). All mutations alter the molecular constitution of Nav1.7 channels, resulting in clinical phenotypes with an approximate penetrance of 100 % [13–36]. About 3/4 of the mutations have been localized to S4, S4/S5 linker, S5 or S6, suggesting pivotal physiological functions of these structures and possible mutation hot spots [66].

**Table 1:** *SCN9A* mutations and Nav1.7 electrophysiology

Mutation	Domain	Activation	Slow Inactivation	Fast Inactivation	Ramp Currents	RMP	Age of onset (yrs)	Reference
		ΔV _half_ (mV)	ΔV _half_ (mV)	ΔV _half_ (mV)				
*I136V*	D1S1	−5.7	−8	n.s.	Increased	Depolarized	9-22	[27, 29, 67]
*Q10R*	D1S1	−5.3	−4.8	n.s.	Unchanged	Unchanged	14	[25]
*Q10K*	D1S1	n.d.	n.d.	n.d.	n.d.	n.d.	0-20	[17]
*I228M*	D1S4	n.s.	+6.8	n.s.	n.d.	Depolarized	32-46	[20]
*F216S*	DIS4	−11.8	−15.6	n.s.	Increased	n.d.	n.d.	[33, 102]
*S211P*	DIS4	−8.2	−15	n.s.	Increased	n.d.	15	[22]
*S241T*	DIS4/S5 linker	−8.4	−12.3	n.s.	Increased	n.d.	2.5-10	[30, 34]
*I234T*	DIS4/S5 linker	−18	−21	n.s.	Increased	n.d.	<1	[14, 103]
*N395K*	DIS6	−7.7	+13	n.s.	n.d.	n.d.	<10	[28]
*V400M*	DIS6	−6.5	n.s.	+7.3	Increased	n.d.	<1	[104, 105]
*G616R*	DIDII loop	n.s.	n.d.	+5.7	Increased	n.d.	24	[81]
*L823R*	DIIS4	−14.6	n.d.	−9.8	n.d.	n.d.	<1	[24]
*I848T*	DIIS4/S5 linker	−13.8	Hypopolarized	n.s.	Increased	Depolarized	4-8	[36, 67, 76]
*L858H*	DIIS4/S5 linker	−13.3	Hyperpolarized	n.s.	Increased	n.d.	4-8	[36]`[76, 106]
*L858F*	DIIS4/S5 linker	−9	n.s.	+3.1	Increased	n.d.	0-2	[38]
*A863P*	DIIS5	−8	n.s.	+10	Increased	Depolarized	Preschool	[31]
*V872G*	DIIS5	−9.3	n.d.	n.s.	Increased	n.d.	5	[89]
*Q875E*	DIIS5	−17.5	−22.8	n.s.	n.d.	n.d.	2	[13, 40]
*Del-L955*	DIIS6	−24	−39	n.s.	Increased	n.s.	15	[16, 21]
*F1449V*	DIIIDIV	−7.6	Hyperpolarized	+4.3	Unchanged	Unchanged	3	[35]
*P1308L*	DIIIS4/S5 linker	−9.6	n.s.	+16.1	Increased	n.s.	2	[23]
*V1316A*	DIIIS5	−9.3	−10	n.s.	Increased	n.d.	9	[18]
*A1632E*	DIVS4/S5 linker	−7	+3.5	+17	Increased	Depolarized	n.d.	[26]
*W1538R*	DIVS2	−8.6	n.s.	n.s.	n.d.	n.d.	3	[19]
*A1632T*	DIVS5	n.s.	n.s.	+8	Unchanged	Unchanged	17	[15]
*A1746G*	DIVS6	−15.6	−32	n.s.	n.d.	n.d.	61	[19]

*n.d* not determined. *n.s* not significant

So far, whole-cell voltage-clamp studies have shown that PE mutations increase sensory neuron excitability through hyper-polarized shifts in activation, depolarized shifts in steady-state inactivation, slowed deactivations and enhanced ramp currents [13–36]. Evidences showed that these electrophysiological properties are altered in a mutation-dependent manner.

Almost all PE-linked *SCN9A* mutant Nav1.7 channels studied so far displayed hyper-polarized activations. Computer simulations suggested that the hyper-polarized shifts in activation were the major determinant for neuron hyperexcitability induced by PE mutations [26, 28]. To its contradictory, a recent study on a well-identified mutation I848T under 35 °C found that the hyperpolarizing shift in activation diminished under this temperature whereas the depolarizing shift of inactivation remained [67]. This finding suggests a need for reassessment of the close relationship between activation shifts and clinical symptoms [15]. The mechanisms through which hyperpolarized shifts in activation contribute to the hyperexcitability of sensory neurons are that the leftward shifts, with or without a change in steady-state inactivation, increase the overlap between activation and inactivation curves. Researches have demonstrated that this increased overlap enhances ramp currents in Nav1.7 mutant cells compared to wild-type cells, leading to depolarized resting membrane potentials (RMP) [68–70]. The depolarized RMP, as a result, moves closer towards the voltage threshold of Nav1.8 (−16 to −21 mV) [71], which is responsible for the major currents during the upstroke of the all-or-none action potentials in sensory neurons [63, 64].

Del-L955 is an in-frame deletion mutation in *SCN9A* that produces large negative shifts in both activation and slow inactivation. When compared with L858F, a mutation which only causes similar extent of negative shifts in activation but not in slow inactivation, Del-L955 showed attenuating effect on the hyperexcitability in sensory neurons. The result indicates that while the increased overlap between activation and slow inactivation renders sensory neurons hyperexcitable, negative shifts in slow inactivation could alter electrophysiological properties of neuron cells in a hypoexcitable direction.

Several mechanisms underlie enhanced ramp currents in mutant Nav1.7 channels. First, the rightward shifts of steady-state inactivation, accompanied by leftward shifts of activation, amplify ramp currents. Consistently, F1449V mutant channel, which depolarizes steady-state inactivation, displays similar ramp currents with wild-type channels [21]. Studies of other mutations in *SCN9A* (S241T, V400M, A863P, etc.) all showed aforementioned alterations in channel properties and increased ramp currents. Second, the onsets of inactivation are slower in mutant channels compared with wild-type channels. Slow closed-state inactivation of the wild-type Nav1.7 has been associated to the production of large ramp currents [61], so a decrease in the rate of onset of closed-state inactivation would possibly enhance subthreshold currents [32]. Third, under physiological condition, deactivation rate (e.g. sodium channels undergo an open-to-close state) is much larger than inactivation rate at negative potential less than -45 mV [72]. The ability of sodium channels to open and reopen depends more on deactivation rather than inactivation process [72]. However in some mutant channels, take L858F for example, at voltage potentials around -70 mV, deactivation rate is much slower than that of wild-type channels. This permits more channel to open and therefore increasing ramp currents [61]. Indeed, L858H, which possesses slower deactivation kinetics than L858F, creates larger ramp currents [32]. The L858 locus seems to play a major role in modulating both deactivation kinetics and production of subthreshold currents.

The enhanced ramp currents lead to higher availability of sodium channels at RMP during activation and fast inactivation [73]. In DRG neurons, because the ramp currents are triggered in the vicinity of resting potentials (−70 to -40 mV) [74, 75], the larger ramp currents in neurons expressing mutant Nav1.7 channels can amplify small depolarizing inputs, which in turn increases the excitability of DRG neurons [76]. Given the fact that Nav1.7 channels are expressed in small, mostly nociceptive sensory neurons, the alterations in activation, slow inactivation, deactivation and ramp currents provide explanations to the amplified pain symptoms observed in PE patients [77].

#### Mutant-specific effects on Nav1.7 electrophysiology

Besides general channelopathies in PE mutant Nav1.7 channels, studies that focused on particular mutations have shed light upon mutant-dependent mechanisms underlying Nav1.7 channelopathies [24, 30, 33, 36]. For instance, several studies have reported that altered charge of Nav1.7 residues can produce functional abnormalities in these channels. Consistently, L858H and L823R introduce an additional positive charge into S4 segment on DII of Nav1.7 channel. Pronounced hyperpolarizations in activation have been observed in both L858H and L823R transfected cells (−13.3 mV and −14.6 mV respectively), raising the possibility that charge alterations may underlie the large negative shifts of activation in these mutations [24]. However, another mutation F216S that substitutes a charged residue with an uncharged one, was also studied and found to produce a shift in activation of −11.8 mV [33]. Therefore addition or reduction of charge may not be a major modulator of Nav1.7 physiology.

The size of amino acid residues of certain loci might also influence Nav1.7 channel functions. Mutation S241T substitutes serine with threonine, both of which have similar biochemical properties and are polar amino acids, except for that threonine possesses a larger side chain relative to serine, suggesting that the physiological alteration in mutant Nav1.7 channels may be affected by the size of the mutant amino acid residues [30].

#### Nav1.8’s effect on neuron excitability

Researchers have observed an interesting dual manifestation of PE, the divergent functional effects of *SCN9A* mutations on sensory neurons and sympathetic neurons. Mutant Nav1.7 channels render DRG neurons hyperexcitable but increase action potential threshold in sympathetic neurons [78]. Disparate to Nav1.7, Nav1.8 channels have a more depolarized voltage dependence of activation (−16 to -21 mV) and primarily underlie the action potential upstrokes [64]. Studies have shown that Nav1.7 channels are expressed at a high level in both sensory neurons and sympathetic neurons [79], while Nav1.8 channels are mostly expressed in sensory neurons [68]. Additionally, when co-expressed with L858H mutant channel in supra cervical ganglion (SCG) neurons, Nav1.8 tend to protect against the hyperexcitability caused by Nav1.7 mutant channels [68]. Thus, different cell backgrounds, in this case the expression levels of Nav1.8, might have impacts that correlate with direct effects of Nav1.7 mutations on neuron cells.

#### Genotype-phenotype correlation

PE harbors complex genotype-phenotype correlations. Multiple studies have demonstrated that different clinical genotypes have varied influences on phenotypes, and that one genotype can produce distinct clinical phenotypes among different individuals.

Clinical symptoms of PE seem to be mutation-dependent. Generally, most PE patients experienced onset of disease by the first decade of life. However, patients in a Taiwan family encountered the onset of PE symptoms at an average age of 9–22 years old, with the progression to involve hands taking longer time than that seen in most of other PE pedigrees [27]. The causative mutation, I136V, is located within S1 segment in the first domain, which has not been proofed to have a dominant contribution in channel gating [27]. It causes a relatively minor shift in activation (−5.7 mV), suggesting that smaller effects on Nav1.7 channels of certain mutations may be associated with a late onset [27]. Another mutation Q10R, also located in S1 segment in the first domain, was reported to produce an even milder shift in activation [25]. Thereby it appears that the loci of mutation could have certain effects on the phenotype through modulating Nav1.7 gating properties. Interestingly, another mutation Q875E, which shifts voltage dependence of activation by −17.5 mV, was identified in a patient whose symptoms occurred at the age of 15 [13]. Thus the relationship between the effects of mutations and clinical phenotypes should not be strictly causal and other underlying factors should be considered and studied.

Multiple studies have suggested that large shifts in activation are related to early onsets of PE and small shifts in activation are related to late onsets of PE. A study on an in-frame deletion mutation Del-L955 found that this mutation produced the largest shift in slow inactivation (−39 mV) studied to date [21]. This strong enhancement of slow inactivation could reduce channel availability, thus attenuating neuron excitability induced by hyperpolarized activation [21]. In consistent, another mutation A863P, also located in the second domain, depolarizes slow inactivation by 10 mV. A patient with A863P was reported to experience disease onset at preschool period [31].

A1632E mutation presents electrophysiological characteristics of both PE (hyperpolarized activation, slowed deactivation and increased ramp currents) and PEPD (impaired fast inactivation) [26]. A1632E resides in the linker between S4 and S5 segments in the fourth domain, which is highly conservative in sodium channels, suggesting an important role of the S4/S5 linker in channel’s electrophysiological functions. In addition, the A1632E is close to M1627K, one of the PEPD mutations; thereby the mutations on physiological continuum can lead to phenotypes combining distinct pain disorders. Another similar mutation A1632T, which introduces less negative charges to a same locus, leads to PE phenotype without PEPD phenotype [15]. Researches have demonstrated that the negative charge introduced by A1632T has an effect of stabilizing fast inactivation thus attenuating the PEPD phenotype [15]. Additionally, I228M was reported to produce distinct clinical phenotypes in 3 different patients (one PE, the other two small fiber neuropathy) with ages of onset ranging from 32–46 years [20]. Whether the phenotypic diversity is attributable to modifier genes or environment factors still needs further study and will provide deeper insight into pathogenesis of PE.

Previous studies has reported that 4 splice variants of Nav1.7 exist in DRG neurons, including neonatal (N)/adult (A) from exon5 and short (S)/long (L) from exon11 [80]. An increase in expression level of Nav1.7AL has been observed in mature rat DRG neurons [81]. As was reported, G616RAL mutation affects Nav1.7 channel gating properties by enhancing excitability, in ways of depolarizing steady-state inactivation. This electrophysiological change is not seen in G616RNS mutant Nav1.7 [81]. However the hyperexcitability distinguishes from other PE mutations in ways that G616R only depolarizes inactivation while most of other PE mutations hyperpolarize activation. Notably, another study on splice variants of Q10R did not find difference of neuron excitability between adult and neonatal splice isoforms [25]. So even if splice variant can explain the variable ages of onset, the validity of using splice variant to explain other PE-linked neuron hyperexcitability requires further investigations.

On systemic level, Nav1.7 channels are also expressed in central nervous system involved in pain perception and emotional integration, including hypothalamus, habenula and amygdala, suggesting contributions of mutant Nav1.7 channels to higher order misinterpretation of pain [14]. Moreover, alterations of electrophysiology in a certain voltage gated ion channel, as reported, can induce compensatory functional alterations in other ion channels [82]. So the broad range of onset age and phenotypic variation could be possibly illuminated by distinguished compensatory mechanisms among individuals.

### Diagnosis

The diagnosis of PE depends on clinical history and physical examinations. Triad of recurrent redness, burning pain, and warmth of the extremities is the diagnostic hallmark of erythromelalgia. A detailed review of patients’ medical history is necessary to provide information about possible factors that might lead to secondary erythromelalgia. A positive or negative family history can further help to confirm familial/sporadic subclassification of PE. Auxiliary tests such as complete blood count, imaging studies and thermograph can be given for exclusion of other differential diagnoses and secondary erythromelalgia. Histological analysis is not regarded as a routine diagnostic method in PE and is usually performed in severe cases because of limited specificity [83]. Reported histological changes in skin biopsy included perivascular lymphocytic inflammation, perivascular edema (arteriolar endothelial cell swelling and enlarged nuclear) and arteriolar smooth-muscle hyperplasia [84]. In some cases, a significant reduction in the number of small fibers might be found [29]. Also, there have been reports about axonal neuropathy in nerve biopsy and neurogenic atrophy in muscle biopsy [14]. Since biologic markers for PE are not established, a full diagnosis of PE should be carefully made upon the combination of clinical exam findings and detection of a mutation on *SCN9A*. In the absence of general guidelines for *SCN9A* mutation testing, genetic tests can be considered in young patients with positive family history and with secondary etiologies excluded [40].

### Differential diagnosis

To differentiate PE from secondary erythromelalgia, clinical conditions (myeloproliferative disorders, neoplasms, rheumatologic diseases), medications (bromocriptine, calcium channel blockers such as nifedipine, felodipine, and nicardipine and topical isopropanol), substances (mushroom/mercury poisoning) or poxvirus (Chinese epidemic of erythromelalgia) should be carefully reviewed [85]. Due to some shared clinical similarities (severe pain and vasomotor disturbances), PE needs to be differentiated from Fabry disease, which is a rare genetic disorder that causes lipid metabolism aberration and is characterized by burning pain and acroparesthesias in the extremities. A decreased plasmatic level of α –galactosidase A is crucial in the diagnosis of Fabry disease [86]. Other differential diagnoses include Raynaud phenomenon, frostbite, vasculitis, cellulitis, erysipelas, dermatitis, osteomyelitis, complex regional pain syndrome, systemic lupus erythematosus (SLE), peripheral neuropathy, arterial or venous insufficiency and gout [40].

### Genetic counseling

Up to now, there are no general guidelines for genetic test on PE mutations. Upon exclusion of secondary etiology, patients with a positive family history can consider genetic test. Genetic test is influential to family planning because probability of an offspring to inherit the same condition is 50 % [40].

### Treatment

Most cases of PE are refractory to pharmacotherapy and the response to pain therapeutics shows a great heterogeneity [42]. Recent studies have shown that therapeutic inefficiency can be explained by *SCN9A*-linked conformational alterations of the binding locus of local anesthetic agents [18, 28]. For this reason, sodium channel blockers that depend on this mechanism usually display limited efficacy. One study estimated that lidocaine only relieves pain symptom in 55 % PE patients [28]. Therapeutic window of lidocaine is also hindered because of potential significant cognitive, motor and cardiac side effects [45]. In some cases, lidociane produced efficacious pain relief, but long term usefulness was limited [87]. A study compared the effect of lidocaine on N395K versus F216S and demonstrated that mutations located in local anesthetic binding site may be an important determinant for the unsatisfactory drug efficacy [28]. Mexiletine, an anti-arrhythmic agent analogous to lidocaine, was reported to greatly improve pain symptoms in some young PE patients [88]. Additionally, a patient carrying V872G mutation showed favorable response to mexiletine with long-lasting improvement of symptoms [89]. Later it was demonstrated that V872G increased use-dependent effect of mexiletine in PE patients, which could be a therapeutic mechanism more important than tonic block effect. An anti-epilepsy drug, carbamazepine (CBZ) which decreases voltage dependence of fast inactivation of sodium channels to more hyperpolarized potentials [90–92] also provided sensitive pain relief in a three-generation family carrying V400M mutation and a two-generation family carrying I848T [35, 93].

In congenital insensitivity to pain (CIP), a loss-of-function inherited neuropathy, patients do not express Nav1.7 channels. But their insensitivity to pain is not related to deficits in cognitive, sensory or motor ability [94]. A complete Nav1.7 knock-out animal model by null mutations did not show dysfunction of other sodium channel within DRG neurons. This indicates that Nav1.7 could be an ideal target for novel pain-relieving drugs [45].

Several novel Nav1.7-selectvie agents are undergoing clinical trials and display desirable efficacy. An oral administrated Nav1.7 channel modulator, PF-05089771, has completed phase II clinical trial of PE [95]. Another Nav1.7 channel blocker, TV-45070 (formerly XEN402), which is administrated through topical approach, has been granted orphan drug for PE [96] and is now undergoing phase II proof-of-concept clinical trial of PE [97]. In addition, the tarantula venom peptides ProTx-I and ProTx-II stand out from other sodium channel blockers in that ProTx-I and ProTx-II shift activation of VGSCs positively while most of other sodium channel blockers modulate inactivation process [98]. Notably, it is reported that ProTx-II selectively inhibited to Nav1.7 and completely prevented activation of evoked C-fiber at concentrations that had little effect on Aβ-fiber conduction [99]. Besides nonspecific or Nav1.7-selectvie sodium channel blockers, an experiment using anti-Nav1.7 sequences via viral delivery was reported to attenuate inflammatory pain in animal models [100], thus providing more possibilities concerning therapeutic approaches.

In addition, other reported topical therapies for PE involve midodrine and botulinum toxin. Thoracic sympathectomy has been reported to have successful clinical outcomes [101]. Besides, non-pharmaceutical treatments include cooling or elevating the affect extremities, avoiding excessive warming and long-time standing or exercise.

### Prognosis

Most PE patients remain refractory to sodium channel blockers and their symptoms progress overtime. Most patients will develop self-mutilating behaviors or tissue damage such as ulceration, necrosis, and gangrene of affected extremities. Patients’ quality of life is greatly compromised and disability is not rare due to intolerable pain, secondary tissue damage or self-mutilating behaviors.

## Conclusion

Since the identification of *SCN9A*, more than 20 PE mutations have been reported. Researches into PE have shed light upon the pivotal role of voltage-gated sodium channels in sensory neurons’ physical properties and have elucidated the underlying pathogenic mechanisms caused by *SCN9A*-linked Nav1.7 mutations. Electrophysiology studies of mutant Nav1.7 channels have presented us a complex yet intriguing network of genotype-channelopathy-phenotype correlation, through looking into which more aspects of the nociception pathways can be clarified. Studies on CIP caused by loss-of-function mutations in Nav1.7 and Nav1.7 knock-out mice models suggest that Nav1.7 is an ideal target for novel pain-relieving drugs. Furthermore, several novel Nav1.7-selectvie agents have showed satisfactory clinical efficacy. In order to delineate an overall picture of this rare and intricate pain disorder, several questions remain to be answered such as whether molecular modulators or non-coding regions of *SCN9A* also contribute to the mechanisms of PE? What explains for the disproportionate relationship between the level of channelopathies and phenotypes? Whether Nav1.7-specific blockers have therapeutic value in all gain-of-function *SCN9A* mutations? What remains elusive may be the keystone to bridge the gap between basic researches to clinical treatments for PE.
